# Low Health Literacy and Mortality in Individuals with Cardiovascular Disease, Chronic Obstructive Pulmonary Disease, Diabetes, and Mental Illness: A 6-Year Population-Based Follow-Up Study

**DOI:** 10.3390/ijerph17249399

**Published:** 2020-12-15

**Authors:** Karina Friis, Anna Aaby, Mathias Lasgaard, Marie Hauge Pedersen, Richard H. Osborne, Helle Terkildsen Maindal

**Affiliations:** 1 DEFACTUM, Central Denmark Region, 8200 Aarhus, Denmark; Mathias.Lasgaard@stab.rm.dk (M.L.); Marie.Pedersen@rm.dk (M.H.P.); 2 Department of Public Health, Section for Health Promotion and Health Services, Aarhus University, 8000 Aarhus, Denmark; aaby@ph.au.dk (A.A.); htm@ph.au.dk (H.T.M.); 3 Centre for Global Health and Equity, Swinburne University of Technology, Melbourne 3025, Australia; rosborne@swin.edu.au; 4 Health Promotion, Steno Diabetes Centre Copenhagen, 2820 Gentofte, Denmark

**Keywords:** health literacy, mortality, cardiovascular disease, diabetes, mental illness

## Abstract

Background: The objective of the study was to examine the impact of health literacy on mortality in the general population and among individuals with cardiovascular disease (CVD), chronic obstructive pulmonary disease (COPD), diabetes, and mental illness. Methods: Data from a large Danish health survey (*n* = 29,473) from 2013 were linked with national mortality registry data to permit a 6-year follow-up. Results: Individuals reporting difficulties in understanding information about health, had higher risk of dying during follow-up (hazard rate (HR) 1.38 (95% CI 1.11–1.73)) compared with those without difficulties. Higher risk was also observed among people reporting CVD (HR 1.47 (95% CI 1.01–2.14)), diabetes (HR 1.91 (95% CI 1.13–3.22)) and mental illness (HR 2.18 (95% CI 1.25–3.81)), but not for individuals with COPD. Difficulties in actively engaging with healthcare providers was not associated with an increase in the risk of dying in the general population or in any of the four long-term condition groups. Conclusions: Aspects of health literacy predict a higher risk of dying during a 6-year follow-up period. Our study serves as a reminder to healthcare organizations to consider the health literacy responsiveness of their services in relation to diverse health literacy challenges and needs.

## 1. Introduction

The persistence of social inequality in life expectancy is a major public health concern [1,2]. One of the mechanisms underlying social inequality in health may be found in the concept of health literacy, which captures the difficulties people may encounter in navigating healthcare systems. Health literacy is defined as the personal competences and situational resources needed for people to access, understand, appraise and use information and services to make health-related decisions. It includes the capacity to communicate, be assertive and act upon their decisions [3]. In addition, health literacy refers to the ways in which services, organizations and systems make health information and resources available and accessible to people according to their health literacy strengths and limitations [4].

There is increasing evidence that low health literacy, mainly captured as health-related reading and numeracy ability (i.e., functional health literacy), is associated with adverse health outcomes [5,6,7,8,9,10,11], including premature death [5,12,13,14,15,16,17,18,19,20,21,22]. Most studies of health literacy and mortality emanate from the United States [5,12,14,15,17,20,22]. Poor health outcomes resulting from health literacy challenges are often the result of a lack of individual abilities when navigating complex healthcare systems [23]. Research from the United States can therefore hardly be generalised to a Western European setting, where healthcare systems differ with regard to health service providers and payment schemes. 

Another limitation of previous research on health literacy and mortality is the frequent use of performance-based health literacy measures that measure health-related reading and numeracy skills [5,12,13,15,17,19,20,21,24,25,26]. Broader and more comprehensive health literacy measures have only rarely been used [14,18,22]. These measures aim to capture specific health literacy needs and challenges, such as a person’s confidence, social resources, and ability to navigate the healthcare system; as such, the latter offer a more accurate description of health literacy. 

The aim of this study was to examine the impact of two key health literacy abilities (namely, the ability to understand information about health and the ability to engage actively with healthcare providers) on the risk of all-cause mortality during a 6-year follow-up period in a large population-based cohort. We hypothesized that individuals with difficulties in these two health literacy dimensions would have an increased risk of dying during follow-up. The study was conducted in the general population and in individuals with four long-term conditions: cardiovascular disease (CVD), chronic obstructive pulmonary disease (COPD), diabetes, and mental illness. These long-term conditions were selected as a recent study has shown that individuals with these conditions reported more difficulties than the general population in understanding health information and actively engaging with healthcare providers [27]. 

## 2. Materials and Methods 

### 2.1. Setting and Baseline Survey Data

The baseline survey data were derived from the “How Are You?” survey conducted in 2013 by the Central Denmark Region, which is one of the five geographical regions in Denmark. The population in the Central Denmark Region has a demographic composition similar to that of the total Danish population [28].

A random sample of 46,354 individuals above the age of 24 years was invited to participate in the survey. In total, 29,473 individuals (63%) participated by answering an online or paper questionnaire. 

### 2.2. Health Literacy 

We used two scales from the nine-scale multidimensional Health Literacy Questionnaire (HLQ) [29]: “Understanding health information well enough to know what to do” and “Actively engaging with healthcare providers”. These two scales cover distinct dimensions of health literacy that are essential for active self-care among people with long-term conditions. The HLQ was translated and underwent validity testing and was found to have robust psychometric properties for use in the Danish population [30]. Each scale comprises five items where respondents answer on a four-point scale: 1 = very difficult, 2 = difficult, 3 = easy and 4 = very easy (“Understanding” α = 0.87 and “Engagement” α = 0.91). The scale scores were calculated as the mean of the five item scores and then standardized to a range between 1 (lowest ability) and 4 (highest ability) to ensure consistency across the response options. Respondents with more than two missing item responses on a scale, were treated as missing data. As a result of this 1961 observations (6.7%) were excluded from the “Understand health information” scale and 1924 observations (6.5%) from the “Actively engage with healthcare providers” scale. Each scale was coded into a binary variable (score ≤ 2) that identified individuals who found it very difficult or difficult to understand health information or actively engage with healthcare providers. 

### 2.3. Measures of Long-Term Conditions

Information about CVD, COPD, diabetes and mental illness was self-reported. In relation to CVD, we included those who answered that they had or had previously had myocardial infarction, angina pectoris or stroke. In relation to COPD, diabetes and mental illness (both short-term and long-term mental illness), we only included those who had the condition at the time of data collection.

### 2.4. Follow-Up Data and Outcome Measures 

All Danish citizens receive a unique personal identification number (CPR number) at birth or immigration. This number is used in all health databases, permitting unambiguous record linkage. Using the CPR number, we linked all survey respondents with person-specific longitudinal register data from the Danish Civil Registration System [31] for a six-year period—from the beginning of 2013 until April 2019. The survey data were merged with registry data to retrieve the exact date of death for all those who died in the follow-up period.

### 2.5. Confounders

Consistent with previous research [5,12,13,14,15,17,18,19,20,21,22], we included the following potential confounders at baseline: gender, age, educational level, ethnic background and cohabitation status. Information on gender, age and ethnic background was collected from registry data, whereas the information on other aspects was self-reported. Educational level was divided into low (1–10 years of education), medium (11–14 years of education) and high (≥15 years of education). In relation to ethnic background, a person was defined as Danish if he or she had at least one parent with Danish citizenship. Cohabitation status was categorized either as living with a partner or not living with a partner. 

Multimorbidity was categorized as 0, 1, 2, 3 or 4+ long-term conditions at baseline (i.e., diabetes, hypertension, CVD, COPD, osteoarthritis, rheumatoid arthritis, osteoporosis, cancer, mental illness, and slipped discs or other back injuries). 

We also included four self-reported measures of health behaviour—smoking, alcohol consumption, physical inactivity and unhealthy diet. Respondents who answered that they smoked on a daily basis were classified as smokers. Respondents were asked how many alcoholic drinks per week they normally drank. High-risk alcohol consumption (yes/no) was categorized in accordance with the Danish Health Authority’s recommendations, i.e., more than 21 drinks weekly for men and 14 drinks for women. Respondents were classified as physically inactive if, during a typical week, they were not physically active on at least one day for a minimum of 30 min. Dietary habits were assessed using the validated Diet Quality Score [32], which classifies diet quality (unhealthy vs. healthy) in relation to cardiovascular risk.

### 2.6. Statistical Data Analysis

Register data on respondents and non-respondents were used to construct a weight to account for differences in selection probabilities and response rates. This was done by Statistics Denmark, using a model-based calibration approach [33]. Data were weighted to represent the population in the Central Denmark Region. 

Cox regression analyses were used to examine if the two health literacy variables were predictors of all-cause mortality during a six-year follow-up period. Outcomes were expressed as hazards ratios (HRs). Survival time was measured in days from the date of invitation to participate in the survey (1 February 2013) to the date of death, emigration or the end of follow-up (30 April 2019) (right censoring), whichever occurred first. Completeness of follow-up on mortality was 99.5% (156/29,473 respondents emigrated).

Regression models were conducted at the general population level and for each of the four long-term condition groups. Model 1 displays unadjusted HRs. Model 2 was adjusted for gender, age, educational level, ethnic background and cohabitation status at baseline. Model 3 was adjusted for the same variables as in model 2 plus self-reported multimorbidity. Finally, model 4 was adjusted for the same variables as in model 3, plus health behaviour. Individuals who found it easy to understand information about health or engage actively with healthcare providers were the reference groups in all of the analyses. STATA version 15 was used for all analyses.

## 3. Results

The mean age of the sample was 52.1 years (SD = 16.3), and the gender distribution was equal (Table 1). In total, 2389 (7.5%) reported CVD, 1214 (3.9%) reported COPD, 1685 (5.5%) reported diabetes and 1577 (6.4%) reported a mental illness. The mean age was lowest among individuals with mental illness (48.1 years) and ranged from 63.6 years to 66.1 years in the three other chronic condition groups. The vast majority of individuals with one of the four long-term conditions also had additional long-term conditions. Compared to the general population, the percentage of individuals who found it difficult to understand information about health or actively engage with healthcare providers was markedly higher in all of the four long-term condition groups. 

In the general population, 6.7% died during the six-year follow-up period (Table 2). The percentage was markedly higher in individuals with CVD (21.3%), COPD (26.3%) and diabetes (18.7%), whereas the mortality rate in individuals with mental illness (7.2%) did not differ from that of the general population. 

Low health literacy, in terms of difficulty understanding information about health, was a predictor of mortality in the general population at follow-up (Table 3). Even after adjusting for sociodemographic factors, multimorbidity and health behaviour at baseline (Model 4) the adjusted HR demonstrated a 1.38 (95% CI 1.11–1.73) higher risk of dying at follow-up compared with individuals who did not have this difficulty. Higher risks were also seen for individuals who reported CVD (HR 1.47 (95% CI 1.01–2.14)), diabetes (HR 1.91 (95% CI 1.13–3.22)) and mental illness (HR 2.18 (95% CI 1.25–3.81)) but not for individuals with COPD (HR 0.71 (95% CI 0.41–1.21)). 

Difficulties in actively engaging with healthcare providers increased the risk of dying during follow-up in the general population (1.35 (95% CI 1.10–1.67)) when adjusted for sociodemographic factors and multimorbidity at baseline (Model 3). However, after further adjustment for health behaviour at baseline (Model 4), an association was not observed (HR 1.19 (95% CI 0.94–1.49)) in the general population. In Model 4, no association was found in any of the four long-term condition groups. 

## 4. Discussion

This study showed that individuals who find it difficult to understand information about health have a 1.4-fold higher mortality risk after 6 years than individuals who do not report difficulties in understanding health information. The increased mortality was observed in the general population as well as among individuals with CVD, diabetes and mental illness. The pathway between low health literacy and mortality is not well understood. One explanation is that individuals with low health literacy do not use healthcare systems effectively; moreover, they experience poorer communication with healthcare professionals and have worse self-care behaviour [23,34,35,36,37]. Differences between individuals with low and high health literacy may also be explained by lack of knowledge and skills, and by differences in attitudinal and motivational factors, such as less information seeking and lower self-efficacy for health-related actions [35,36,37], lower use of preventive screening [38] and later presentation of illness among individuals with low health literacy [34,39].

There was no association between health literacy and mortality in individuals with COPD. It is possible that the biological process of COPD outweighs the potential influence of health literacy. The main cause of COPD in Denmark is smoking [40], which often carries with it substantial feelings of shame, guilt or stigma, which may impede relationships with healthcare professionals. Perhaps many people with COPD understand health information well enough to know what to do; however, the health-related impact of smoking is in some cases irreversible at baseline as smoking cessation is difficult to manage. Furthermore, the chronic and highly complex nature of COPD means that patients engage for many years with health professionals and receive a great deal of information and support. It is likely that the pathological process and physical deterioration are the strongest determinants of prognosis of COPD regardless of the health literacy level. To the best of our knowledge, only one other study has investigated the impact of functional health literacy on mortality risk in individuals with COPD [24]. In a Spanish study of 296 COPD patients, low health literacy was not found to predict all-cause mortality in the following year. However, a trend was seen in that those with low health literacy had a worse survival expectation [24]. 

It is difficult to compare the findings across studies due to the use of different measurement tools. Most previous tools used health-related reading and numeracy tests [5,12,13,15,17,19,20,21,24,25,26]. However, in accordance with our findings, most studies using these tests found that low health literacy predicted higher mortality rates [5,12,13,15,17,19,20,21]. A few studies analysed the association between health literacy and mortality using a subjective health literacy measure; however, the studies were limited to patients with heart failure in the United States [14,18,22]. 

The important strengths of this study include that it is a population-based registry-based study with almost complete follow-up. The study was large, with a reasonable number of events. This permitted reasonably precise estimates of the effects of two aspects of health literacy, including after adjustment for comorbidities. A major limitation of the study, however, is that we only measured two aspects of the multidimensional concept of health literacy. We selected two of the nine most disparate scales of the Health Literacy Questionnaire; however, other elements are also likely to be important and it is known that individuals can have different patterns of health literacy strengths and weaknesses [41]. 

A limitation of the present study, inherent in any population-based survey of health literacy, is that individuals with the lowest levels of health literacy and/or with the poorest health status may struggle to complete the questionnaire and may, therefore, be less likely to respond to the survey. About 7% of the respondents to the questionnaire did not answer the HLQ questions. Those who did not answer these questions were older, more of them did not have a Danish ethnic background and they had lower levels of education. Even though data were weighted to represent the general population, the survey may be subject to a non-response bias that overestimates the population’s health literacy levels, potentially weakening associations with mortality. 

## 5. Conclusions

To conclude, our study may serve as a reminder to healthcare organizations to consider the health literacy responsiveness of their services in relation to diverse health literacy challenges and needs [42,43]. Approaches to improve health literacy responsiveness and ultimately decrease mortality may include easy access to information and services, more effective provider–patient communication, better patient education materials, individualized self-care support for those with health literacy challenges, relevant postgraduate professional training, and more efficient collaboration across healthcare providers in communicating information and targeting services to individual needs. Our results indicate that there is much to gain from a health literary approach to inequality in life expectancy.

## Figures and Tables

**Table 1:** Participant characteristics at baseline (2013).

	General Population *n* = 29,473	Cardiovascular Disease *n* = 2389 (7.5%)	Chronic Obstructive Pulmonary Disease *n* = 1214 (3.9%)	Diabetes *n* = 1685 (5.5%)	Mental Illness *n* = 1577 (6.4%)
	*n* (% ^1^)	*n* (% ^1^)	*n* (% ^1^)	*n* (% ^1^)	*n* (% ^1^)
**Gender**					
Women	15,448 (50.6)	918 (41.2)	584 (50.8)	731 (45.1)	959 (58.7)
**Age (mean (SD))**	52.1 (16.3)	65.7 (14.0)	66.1 (12.2)	63.6 (13.4)	48.2 (14.7)
**Level of education**					
Low (1–10 years)	5507 (18.6)	736 (33.7)	440 (37.6)	529 (34.1)	389 (25.9)
Medium (11–14 years)	14,718 (50.2)	1147 (49.1)	571 (49.1)	790 (48.9)	731 (47.0)
High (≥15 years)	8319 (31.2)	409 (17.2)	160 (13.3)	282 (17.1)	407 (27.1)
**Cohabitation status**					
Living alone	6657 (30.3)	690 (38.5)	424 (45.9)	479 (38.4)	982 (52.8)
**Ethnic background**					
Not Danish	1073 (6.4)	71 (5.0)	17 (2.0)	52 (5.0)	125 (10.7)
**Number of (additional) long-term conditions**					
0	14,847 (54.5)	632 (26.9)	251 (20.0)	300 (18.6)	636 (43.7)
1	7450 (24.1)	708 (27.9)	350 (27.7)	578 (32.6)	392 (23.7)
2	3936 (12.4)	562 (22.9)	299 (25.1)	444 (24.7)	263 (15.1)
3	1749 (5.5)	309 (13.5)	175 (13.7)	225 (14.6)	170 (9.8)
4+	989 (3.5)	178 (8.8)	139 (13.5)	138 (9.5)	116 (7.7)
**Health literacy**					
Difficult to understand information about health	1037 (4.2)	181(9.1)	94 (9.6)	121 (9.3)	165 (11.9)
Difficult to engage actively with healthcare providers	1801 (6.9)	217 (11.1)	126 (13.1)	133 (9.3)	263 (17.7)

SD = Standards deviation ^1^ Weighted data.

**Table 2:** Mortality at the end of 6-year follow-up in relation to health literacy in the general population and by long-term condition.

	General Population *n* = 29,473	Cardiovascular Disease *n* = 2389	Chronic Obstructive Pulmonary Disease *n* = 1214	Diabetes *n* = 1685	Mental Illness *n* = 1577
	**% (95% CI)**	**% (95% CI)**	**% (95% CI)**	**% (95% CI)**	**% (95% CI)**
**Total**	6.7 (6.4–7.0)	21.3 (19.4–23.3)	26.3 (23.5–29.4)	18.7 (16.6–21.1)	7.2 (5.9–8.8)
**Understand information about health**					
Easy	5.5 (5.2–5.8)	18.0 (16.1–20.1)	25.2 (22.2–28.6)	16.1 (13.9–18.5)	6.0 (4.7–7.7)
Difficult	16.8 (14.4–19.5)	37.4 (29.6–45.8)	22.5 (15.3–31.8)	33.3 (24.4–43.5)	13.9 (9.4–20.1)
**Actively engage with health care providers**					
Easy	5.8 (5.5–6.1)	19.0 (17.0–21.1)	24.6 (21.5–27.9)	17.1 (14.9–19.6)	6.4 (5.0–8.1)
Difficult	8.6 (7.2–10.2)	28.2 (21.6–36.0)	25.8 (18.0–35.4)	24.8 (17.2–34.3)	9.7 (6.5–14.2)

**Table 3:** Hazard ratio of dying at 6 year follow-up in relation to health literacy in the general population and in individuals with different long-term conditions.

	Model 1 Unadjusted HR (95% CI)	Model 2 ^1^ Adjusted for Sociodemographic Factors HR (95% CI)	Model 3 ^2^ Adjusted for Sociodemo- graphic Factors and Multimorbidity at Baseline HR (95% CI)	Model 4 ^3^ Adjusted for Sociodemo- graphic Factors, Multimorbidity and Health Behaviour at Baseline HR (95% CI)
**General population** (*n* = 29,473)				
Difficult to understand information about health ^4^	3.29 (2.75–3.94)	1.89 (1.57–2.28)	1.75 (1.45–2.10)	1.38 (1.11–1.73)
Difficult to engage actively with healthcare providers ^5^	1.51 (1.25–1.83)	1.54 (1.24–1.90)	1.35 (1.10–1.67)	1.19 (0.94–1.49)
**Cardiovascular disease** (*n* = 2389)				
Difficult to understand information about health ^4^	2.38 (1.76–3.22)	1.69 (1.25–2.27)	1.55 (1.15–2.11)	1.47 (1.01–2.14)
Difficult to engage actively with healthcare providers ^5^	1.61 (1.16–2.24)	1.64 (1.17–2.28)	1.46 (1.03–2.05)	1.38 (0.93–2.07)
**Chronic obstructive pulmonary disease** (*n* = 1214)				
Difficult to understand information about health ^4^	0.89 (0.57–1.39)	0.90 (0.56–1.43)	0.80 (0.49–1.30)	0.71 (0.41–1.21)
Difficult to engage actively with healthcare providers ^5^	1.04 (0.68–1.57)	1.08 (0.70–1.68)	0.97 (0.61–1.52)	0.91 (0.56–1.47)
**Diabetes** (*n* = 1685)				
Difficult to understand information about health ^4^	2.36 (1.59–3.51)	2.06 (1.36–3.13)	1.99 (1.34–2.96)	1.91 (1.13–3.22)
Difficult to engage actively with healthcare providers ^5^	1.55 (1.00–2.41)	1.75 (1.09–2.81)	1.66 (1.05–2.62)	1.20 (0.66–2.17)
**Mental illness** (*n* = 1577)				
Difficult to understand information about health ^4^	2.44 (1.50–3.96)	1.94 (1.14–3.30)	2.03 (1.19–3.45)	2.18 (1.25–3.81)
Difficult to engage actively with healthcare providers ^5^	1.55 (0.95–2.51)	1.58 (0.92–2.70)	1.46 (0.82–2.58)	1.63 (0.94–2.84)

^1^ Adjusted for gender, age, educational level, ethnic background, and cohabitation status. ^2^ Adjusted for gender, age, educational level, ethnic background, cohabitation status, and multimorbidity. ^3^ Adjusted for gender, age, educational level, ethnic background, cohabitation status, multimorbidity, daily smoking, alcohol consumption, dietary habits, and physical activity. ^4^ Reference group = easy to understand information about health. ^5^ Reference group = easy to engage actively with healthcare providers. HR = Hazard ratio; CI = confidence interval.
